# A Comparison of Synthetic Osmotic Dilators and Pharmacologic Agents for Cervical Ripening in Induction of Labor: A Systematic Review and Meta‐Analysis

**DOI:** 10.1111/jmwh.70017

**Published:** 2025-09-08

**Authors:** Gi Wook Ryu, Sun‐Young Park

**Affiliations:** ^1^ Department of Nursing Hansei University Gunpo South Korea; ^2^ College of Nursing Research Institute of Nursing Innovation, Kyungpook National University Daegu South Korea

**Keywords:** cervical ripening, induction of labor, meta‐analysis, prostaglandins, synthetic osmotic dilator, systematic review

## Abstract

**Introduction:**

Given the rising number of studies on synthetic osmotic dilators, there is a lack of comprehensive reviews for their use compared with other commonly used cervical ripening methods. This study aimed to examine the maternal and neonatal safety and efficacy in cervical ripening and labor induction using synthetic osmotic dilators compared with pharmacologic agents (prostaglandin E_1_, prostaglandin E_2_, oxytocin) for labor induction.

**Methods:**

A systematic review and meta‐analysis of randomized controlled trials (RCTs) and cohort studies was conducted, using MEDLINE, Embase, CINAHL, and Cochrane Library databases search. Two reviewers independently screened studies and assessed the risk of bias with Risk of Bias 2 and Risk Of Bias In Nonrandomized Studies ‐ of Interventions tools. Relative risks (RRs) and mean differences (MDs) were calculated with 95% CIs.

**Results:**

Eleven studies (8 RCTs, 3 cohort; 2355 participants) showed no statistically significant differences in safety outcomes between synthetic osmotic dilators and pharmacologic agents, including maternal infection (RR, 1.27), postpartum bleeding (RR, 0.87), neonatal infection (RR, 1.19), low Apgar scores (RR, 0.74), and admission to neonatal intensive care unit (RR, 1.06) (all *P* > .05). Efficacy outcomes were comparable for vaginal birth rates (RR, 0.98) and Bishop score changes (MD, 0.0) (both *P* > .05). Synthetic osmotic dilators reduced uterine hyperstimulation (RR, 0.45) and digestive symptoms (RR, 0.15) but required more artificial rupture of membrane (RR, 1.57) (all *P* < .05).

**Discussion:**

Synthetic osmotic dilators are a safe, effective, and viable option for labor induction, reducing maternal risks of uterine hyperstimulation. These findings have implications for incorporating the clinical use of synthetic osmotic dilators for cervical ripening into international guidelines. As evidence supports their efficacy and safety, educating nurses and midwives in the use of synthetic osmotic dilators for labor induction is required.

## INTRODUCTION

Induction of labor has been steadily increasing, reaching 20% to 24% of full‐term births in the United Kingdom and the United States.^1^, ^2^ The reasons for the rise in labor induction include the increasing prevalence of comorbidities among pregnant women—such as obesity, hypertension, and diabetes—and the desire to reduce maternal and fetal risks and discomfort associated with childbirth, which has led to an increasing preference for scheduling the timing of birth.^3^, ^4^, ^5^, ^6^, ^7^

Adequate cervical ripening is critical for successful induction of labor, particularly when the Bishop score is less than 6.^8^ Available cervical ripening methods include pharmacologic agents (prostaglandin E_2_ [PGE_2_; dinoprostone], prostaglandin E_1_ [PGE_1_, misoprostol], and oxytocin) ^9^, ^10^, ^11^ and mechanical methods (Foley catheters, double balloon catheters, laminaria tents, and synthetic osmotic dilators).^12^, ^13^

**QUICK POINTS**

✦ With the rising rates of labor induction and growing evidence supporting the use of synthetic osmotic dilators, it is essential for midwives and nurses to be informed about emerging cervical ripening methods.
✦ Synthetic osmotic dilators show comparable maternal and neonatal safety and efficacy to pharmacologic agents (eg, prostaglandin E_1_, prostaglandin E_2_, oxytocin) for cervical ripening.
✦ Synthetic osmotic dilators are associated with a lower incidence of uterine hyperstimulation and digestive symptoms compared with pharmacologic agents during labor induction.
✦ Synthetic osmotic dilators are effective alternatives when pharmacologic agents are contraindicated for labor induction in patients with a previous cesarean birth.

PGE‐based agents are the most common cervical ripening method.^9^, ^12^, ^14^ PGE_1_ facilitates cervical softening by acting on the intracellular matrix, promoting collagen fibril breakdown,^15^ whereas PGE_2_ promotes cervical ripening by altering the extracellular matrix, softening the cervix, relaxing the cervical smooth muscle, and stimulating uterine contractions.^16^ However, PGE agents are contraindicated in women with previous cesarean birth due to the increased risk of uterine hyperstimulation and rupture,^7^, ^17^ and continuous monitoring of uterine activity and fetal condition is required during their use.^2^ Oxytocin is typically used when the cervix is favorable (Bishop score ≥7‐8) and primarily acts to stimulate uterine contractions.^18^

Mechanical ripening methods, such as cervical balloons or synthetic osmotic dilators, are available for labor induction in women with a history of cesarean birth,^14^ due to their lower risk of uterine hyperstimulation compared with pharmacologic approaches.^2^ Foley balloon catheters are the most widely used mechanical method.^9^, ^12^, ^14^ They function by inflating a balloon with 30 to 60 mL sterile fluid, which applies direct pressure to the cervix, stimulating endogenous prostaglandins release and promoting cervical ripening.^10^, ^11^, ^19^ Compared with PGE‐based ripening methods, Foley balloon catheters have advantages in their low cost (Foley balloon: US$3866 versus PGE2 $3,606) and stability at room temperature (unlike PGEs, which require refrigeration storage).^11^, ^20^, ^21^

Among synthetic osmotic dilators, Dilapan (made of polyacrylonitrile) was introduced in the early 1990s,^22^ whereas Dilapan‐S and Dilasoft, second‐generation dilators that are made of Aquacryl hydrogel, were introduced after 2015 and are designed to gradually expand by absorbing moisture from the cervical tissue.^9,^ ^23^ Dilasoft was primarily developed for the Japanese market and is primarily sold there, whereas Dilapan‐S received Food and Drug Administration (FDA) approval in 2015 for cervical ripening in the third trimester in the United States and The Conformité Européene (CE) certification in the European Union.^9^, ^14^ Since receiving FDA approval, numerous clinical trials have reported that synthetic osmotic dilators are safe and effective for cervical ripening and induction of labor.^15^, ^24^

When Dilapan‐S is used, 3 to 5 rods are inserted into the endocervical canal, with each dilator having a preattached marker string for localization and removal.^15^, ^23^ As the dehydrated rods absorb fluid, they expand radially over 4 to 6 hours (up to 24 hours if needed), exerting consistent pressure to promote cervical dilation, stimulate the release of endogenous prostaglandins, and facilitate cervical softening.^23^

Compared with pharmacologic agents, synthetic osmotic dilators have been associated with lower rates of uterine hyperstimulation, greater maternal satisfaction, and reduced pain during cervical ripening.^15^ A study comparing synthetic osmotic dilators and vaginal PGE_2_ demonstrated cost neutrality between the 2 methods (synthetic osmotic dilators: $4829 vs PGE_2_: $4837); synthetic osmotic dilators reduced midwife time by 146 minutes and obstetrician time by 11 minutes, respectively.^25^

Compared with Foley balloon catheters, synthetic osmotic dilators have practical benefits such as no protrusion from the introitus and no requirement to maintain balloon tension.^26^ These advantages can decrease maternal pain and increase comfort, including better sleep quality, relaxation, and daily activity levels.^26^ Additionally, synthetic osmotic dilators present comparable maternal and neonatal outcomes and efficacy for changes in Bishop scores.^26^

International guidelines, such as those from the National Institute for Health and Care Excellence (NICE), World Health Organization (WHO), and American College of Obstetricians and Gynecologists (ACOG), recommend various mechanical methods such as Foley catheters and double balloon catheters for cervical ripening, but guidance for synthetic osmotic dilators is lacking.^2^, ^7^, ^20^, ^27^ Both the NICE^2^ and ACOG^20^ guidelines recommend PGE‐based methods as the first‐line option for cervical ripening in induction of labor, with Foley balloon catheters^2^, ^20^ or osmotic cervical dilators^2^ suggested as alternative ripening methods. The NICE guideline suggests that mechanical methods require less‐intensive monitoring for uterine hyperstimulation compared with pharmacologic methods and recommends considering mechanical methods when preferred by the patient.^2^ The WHO guideline also endorses both PGE‐based methods and Foley balloon catheters as effective options for cervical ripening, and it specifically recommends the simultaneous combination of a Foley balloon catheter and oxytocin in cases with a high risk of uterine hyperstimulation or in women with a history of previous cesarean birth.^7^, ^27^ Although mechanical ripening methods, including synthetic osmotic dilators, were evaluated by a Cochrane review, the evidence on synthetic osmotic dilators is inconclusive regarding efficacy and safety.^11^

Given the differences in indications, costs, and safety profiles among cervical ripening methods, a comprehensive assessment of these methods, particularly those newly introduced to clinical settings compared with traditional cervical ripening methods, is essential to strengthen the evidence supporting their use. However, to date, rigorous evidence that comprehensively compares the efficacy and safety of synthetic osmotic dilators with other cervical ripening methods remains limited.^28^ Although an increasing number of studies^15^, ^22^, ^24^, ^29^, ^30^, ^31^, ^32^, ^33^, ^34^, ^35^, ^36^ have examined synthetic osmotic dilators compared with pharmacologic agents, which are commonly used ripening methods, comprehensive evaluations remain scarce. Furthermore, despite the reported advantages of synthetic osmotic dilators over Foley balloon catheters, only 2 studies^26^, ^34^ have compared their safety and efficacy outcomes, indicating that more studies need to be accumulated to precisely compare the outcomes of both methods.

To fill these gaps, this study aimed to compare synthetic osmotic dilators with pharmacologic agents for cervical ripening in terms of maternal and neonatal safety as well as efficacy in cervical ripening and labor induction, including success of vaginal birth, Bishop score changes, need for additional ripening or interventions (oxytocin, artificial rupture of membranes [AROM], vacuum or forceps vaginal birth), and induction‐to‐birth time, using meta‐analysis methods.

## METHODS

This systematic review and meta‐analysis evaluated the safety and efficacy of synthetic osmotic dilators for labor induction, comparing them with pharmacologic agents. This review adhered to the guidelines of the Preferred Reporting Items for Systematic Reviews and Meta‐Analyses (PRISMA)^37^ (Supporting Information: Appendix S1). This review's protocol was registered in the PROSPERO (registration no: CRD42024587953).

### Search Sources and Strategies

MEDLINE, Embase, CINAHL, and the Cochrane Library were searched for literature published from their inception to August 27, 2024, by an author (S.Y.P). Search terms were related to *pregnancy*, *induction of labor*, and *synthetic osmotic dilator* (Supporting Information: Appendix S2). Literature identified from the database search was transferred to a reference manager (EndNote, version 21), and duplicates were removed using Bramer's method.^38^ Two authors (G.W.R and S.Y.P) independently reviewed the titles and abstracts of the selected studies, followed by one author (S.Y.P) reviewing the full‐text of the eligible studies. In cases of disagreement during the study selection process, the authors discussed and reached a consensus to determine the final selection.

### Study Selection

We searched and selected studies based on the PICO framework (Population‐Intervention‐Comparison‐Outcome).^37^ We included studies of pregnant individuals requiring induction of labor, without restrictions on gestational age or history of previous cesarean birth (Population), comparing synthetic osmotic dilators alone or in combination with pharmacologic agents (PGE_2_, PGE_1_, oxytocin) (Intervention) versus pharmacologic agents alone (Comparison). Outcomes included maternal safety (infection, uterine hyperstimulation, postpartum bleeding, digestive symptoms), neonatal safety (infection, Apgar scores, neonatal intensive care unit [NICU] admission, perinatal death), and efficacy in cervical ripening and induction of labor (vaginal birth rates, Bishop score changes, and additional interventions including oxytocin administration, instrumental vaginal birth [vacuum or forceps], and AROM).

Eligible study designs included randomized controlled trials (RCTs), non‐RCTs, and cohort studies comparing the safety and efficacy of synthetic osmotic dilators with pharmacologic agents, without publication time restriction. We excluded studies about abortions, nonhuman studies (eg, animal studies), nonoriginal studies (eg, reviews, editorials, letters, and opinions), non‐English publications, and conference abstracts.

### Risk of Bias Assessment

Two independent reviewers (G.W.R. and S.Y.P.) assessed the risk of bias for the selected studies using different assessment tools based on study design.

RCTs were assessed using the Cochrane Risk of Bias 2 (ROB 2) tool, which evaluates bias arising from the randomization process, deviations from intended interventions, missing outcome data, measurement of outcomes, and selection of reported results.^39^ Each domain and the overall risk of bias for a study were evaluated as *low risk*, *high risk*, or *some concerns* using the ROB2 algorithm.^39^

Cohort studies were assessed using the Risk Of Bias In Nonrandomized Studies ‐ of Interventions (ROBINS‐I) tool,^40^ which evaluates bias arising from confounding, selection of participants, classification of interventions, deviations from intended interventions, missing data, measurement of outcomes, and selection of reported results.^40^ Each domain and the overall risk of bias were scored according to the ROBINS‐I algorithm as *low*, *moderate*, *serious*, or *critical* risk of bias.^40^ Risk of bias assessment results were visualized using the Risk‐Of‐Bias Visualization tool.^41^

### Data Extraction

One author (S.Y.P) developed a data extraction template including author of study, publication year, study location, study objective and design, patient characteristics, intervention and comparison procedures, follow‐up period, and the outcomes for safety and efficacy. The same author (S.Y.P) extracted the data from the selected studies using the developed template, and another author (G.W.R) reviewed and checked the extracted data to ensure accuracy.

### Data Synthesis and Analysis

The meta‐analysis was conducted using Review Manager software (version 5.4; Copenhagen, Denmark).^42^ For dichotomous data, relative risks (RRs) with 95% CIs were calculated using random‐effects models to account for heterogeneity in outcome definitions and study designs across studies.^43^ For continuous outcomes, mean differences (MDs) with 95% CIs were calculated using random‐effects models to account for study variations.^43^ Forest plots were generated to visualize the overall patterns of results and heterogeneity among studies, displaying RR, MDs, and their respective 95% CIs for each included study. The subgroup analyses were conducted based on evaluation outcomes and comparators.

Study heterogeneity was assessed using the *I^2^* statistic developed by Higgins et al.^44^ An *I^2^* value less than 25% indicated low, a value between 25% and 75% indicated moderate, and a value greater than 75% indicated high heterogeneity among the selected studies’ outcomes.

Publication bias was assessed through funnel plot symmetry and Egger's test, which was performed only when there were 10 or more studies, using R version 4.4.1.^43^, ^45^ Funnel plots were generated using the *funnel* function, with odds ratios (effect sizes) on the x‐axis and SEs (precision) on the y‐axis, where symmetrical distribution indicates minimal publication bias.^43^ The *metabias* function in the *meta* package was used for Egger's test,^46^ with a *P* < .05 indicating significant publication bias.^45^

## RESULTS

### Study Selection

**Figure 1:**
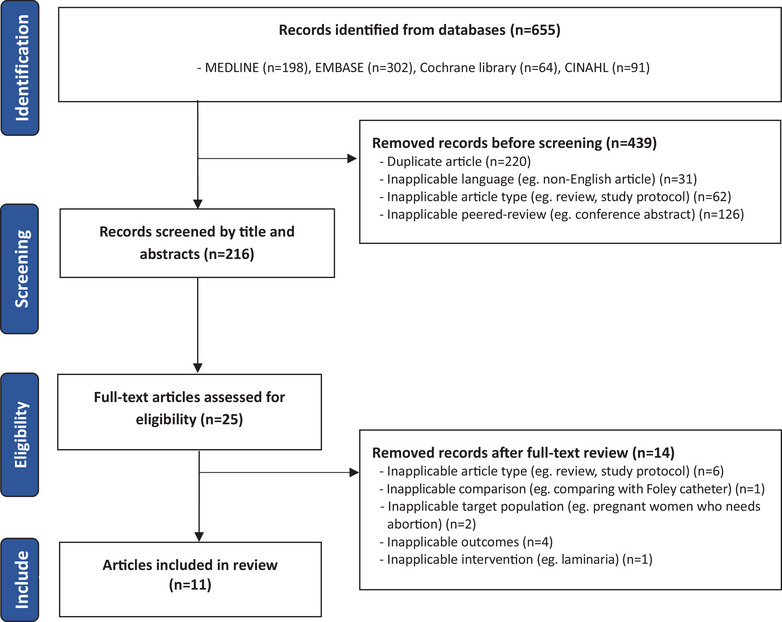
Search and Selection Process of Studies

Figure 1 displays the study search and selection process. Of the 655 screened titles and abstracts, 11 studies with 2355 pregnant women (mean ± SD, 214 ± 186 women; range, 39‐672) at 34 to 41 weeks’ gestation were included in this review.^15^, ^22^, ^24^, ^29^, ^30^, ^31^, ^32^, ^33^, ^34^, ^35^, ^36^

Table 1 summarizes the characteristics of the selected studies. Most studies (81.8%) included both nulliparous and multiparous women.^15^, ^22^, ^24^, ^29^, ^31^, ^32^, ^33^, ^34^, ^36^ Studies were published between 1992 and 1998 (54.5%)^22^, ^29^, ^31^, ^32^, ^35^, ^36^ and 2018 and 2022 (45.5%),^15^, ^24^, ^30^, ^33^, ^34^ with none from 1999 to 2017. Studies published before 1998 primarily used the first‐generation Dilapan, and no studies were published between 1999 and 2017; clinical studies on Dilapan‐S, which received FDA approval in 2015, have been published from 2017 onward.

**Table 1:** Characteristics of Selected Studies (N = 11)

		Population					Intervention				Comparator			
First Author, Published Year (Location)	Study Design	Gestation, wk	Parity	Bishop Score	Previous Cesarean Birth	Clinical Setting (Outpatient vs Inpatient)	Induction Method	Sample Size	No. of Applied Dilapans	Duration, h	Induction method	Sample size	Duration, h	Additional Procedure^a^
Chua, 1997^29^ (Singapore)	RCT	(Mean) Intervention 39.1, comparator 38.9	Nulliparous, multiparous	≤5	NR	Inpatient	Synthetic osmotic dilators (Dilapan)	90	≤4	12	Intracervical PGE_2_ gel 0.5 mg	95	12	Oxytocin and AROM after 12 h
Crosby, 2018^30^ (Ireland)	Cohort	≥41	Nulliparous	≤6	NR	Inpatient	Synthetic osmotic dilators (Dilapan‐S)	26	(Mean) 2.6 (range: 1‐4)	24	Intravaginal PGE_2_ pessary 10 mg	26	≤24	PGE_2_ gel after 24 h, AROM
Gavara, 2022^15^ (United States)	RCT	37	Nulliparous, multiparous	<6	NR	NR	Synthetic osmotic dilators (Dilapan‐S)	151	NR	12	Oral PGE_1_ 25 mcg, up to 6 dose every 2 h	152	12	Oxytocin and AROM after 12 h
Hibbard, 1998^31^ (United States)	RCT	≥34	Nulliparous, multiparous	≤5 (modified Bishop score)	Excluded history of cesarean birth	Inpatient	Synthetic osmotic dilators (Dilapan) and intracervical PGE_2_ gel 0.5 mg after Dilapan application for 12 h	22	3‐6	6	Intracervical PGE_2_ gel 0.5 mg	17	≤18	PGE_2_ gel, oxytocin after 12 h, AROM
Gilson, 1996^22^ (United States)	RCT	(Mean) Intervention 38.2, comparator 38.4	Nulliparous, multiparous	≤4	Excluded history of 2 or more cesarean births	NR	Synthetic osmotic dilators (Dilapan) and oxytocin 1 mU/mL (increased every 30 min after Dilapan application for 12 h	112	Maximum number applied	12	Oxytocin 1 mU/mL (increased every 30 min	128	24	NR
Gupta, 2022^24^ (United Kingdom)	RCT	≥37	Nulliparous, multiparous	Not restricted	Included history of cesarean birth	NR	Synthetic osmotic dilators (Dilapan‐S)	337	≤5	12‐24	Intravaginal PGE_2_ pessary 10 mg	335	≤32	Oxytocin, AROM
Krammer, 1995^32^ (United States)	RCT	≥37	Nulliparous, multiparous	≤8	NR	NR	Synthetic osmotic dilators (Dilapan)	224	Maximum number applied	6	Intracervical PGE_2_ gel 0.5 mg	219	6	Oxytocin after 6 h
Maier, 2018^33^ (Germany)	Cohort	≥41	Nulliparous, multiparous	≤6	Included history of cesarean birth	Inpatient	Synthetic osmotic dilators (Dilapan‐S)	33	≤5	≤24	Intracervical PGE_2_ gel 1 mg and increased up to 3 mg	49	≤24	Additional Dilapan after 12 h, oxytocin and AROM after 24 h
Pekarev, 2022^34^ (Russia)	Cohort	≥37	Nulliparous, multiparous	≤6	NR	NR	Synthetic osmotic dilators (Dilapan‐S)	50	NR	≤12	Intracervical PGE_2_ gel 0.5 mg	50	6	NR
Roztocil, 1998^35^ (Czech Republic)	RCT	≥36	Nulliparous, multiparous	≤5	Excluded women with uterine scar(s)	Outpatient and inpatient	Synthetic osmotic dilators (Dilapan‐S)	82	4	14	Intracervical PGE_2_ gel 0.5 mg	83	14	PGE_2_ after 14 hours and AROM
Sanchez‐Ramos, 1992^36^ (United States)	RCT	(Mean) Intervention 39.2, comparator 39.9	Nulliparous, multiparous	≤6	Excluded women with uterine scar(s)	Outpatient	Synthetic osmotic dilators (Dilapan)	36	Maximum number applied	8‐12	Intracervical PGE_2_ gel 4 mg and additional PGE_2_ gel 4 mg	38	first: 8‐12; second: 4‐6	Oxytocin after 8‐16 h, AROM, additional Dilapan

Abbreviations: AROM, artificial rupture of membranes; NR, not reported; PGE_1_, prostaglandin 1; PGE_2_, prostaglandin 2; RCT, randomized controlled trial.

a Additional procedures were performed when there was no cervical dilation or labor progression after the intervention/comparator procedure.

Research was conducted in the United States (n = 5),^15^, ^22^, ^31^, ^32^, ^36^ Europe (n = 4),^24^, ^30^, ^33^, ^35^ Singapore (n = 1),^29^ and Russia (n = 1).^34^ Most studies (72.7%)^15^, ^22^, ^24^, ^29^, ^31^, ^32^, ^35^, ^36^ were RCTs; 27.3% were cohort studies.^9^, ^30^, ^34^

Regarding previous cesarean birth, 2 studies^13^, ^24^ explicitly included women with a history of cesarean birth; 4 studies^22^, ^31^, ^35^, ^36^ excluded women with uterine scars or history of cesarean birth; and 6 studies^15^, ^22^, ^29^, ^30^, ^32^, ^34^ did not report this information. In terms of the cervical ripening application setting, 4 studies^13^, ^29^, ^30^, ^31^ specified inpatient care; one study^36^ was conducted exclusively in an outpatient setting; one study^35^ included both inpatient and outpatient populations; and 5 studies^15^, ^22^, ^24^, ^32^, ^34^ did not report the care setting.

Six of 11 studies^24^, ^29^, ^30^, ^31^, ^33^, ^35^ applied 3 to 7 dilators per patient for 6 to 24 hours, 3 studies^22^, ^32^, ^36^ applied the maximum number of dilators without specifying the exact number used, and 2 studies^15^, ^34^ did not report the number of dilators used. Nine studies^24^, ^29^, ^30^, ^31^, ^32^, ^33^, ^34^, ^35^, ^36^ used intracervical and/or intravaginal PGE_2_ as comparators, and a single study used oral PGE_1_ (misoprostol)^15^ or intravenous oxytocin.^22^ Two studies compared combinations of synthetic osmotic dilators with pharmacologic agents versus these agents alone; specifically, oxytocin^22^ and PGE_2_.^31^ Most studies (82%)^15^, ^24^, ^29^, ^30^, ^31^, ^32^, ^33^, ^35^, ^36^ implemented additional procedures (oxytocin, AROM, or further PGE_2_) when initial treatment was unable to achieve cervical ripening or labor induction.

### Risk of Bias Assessment

**Figure 2:**
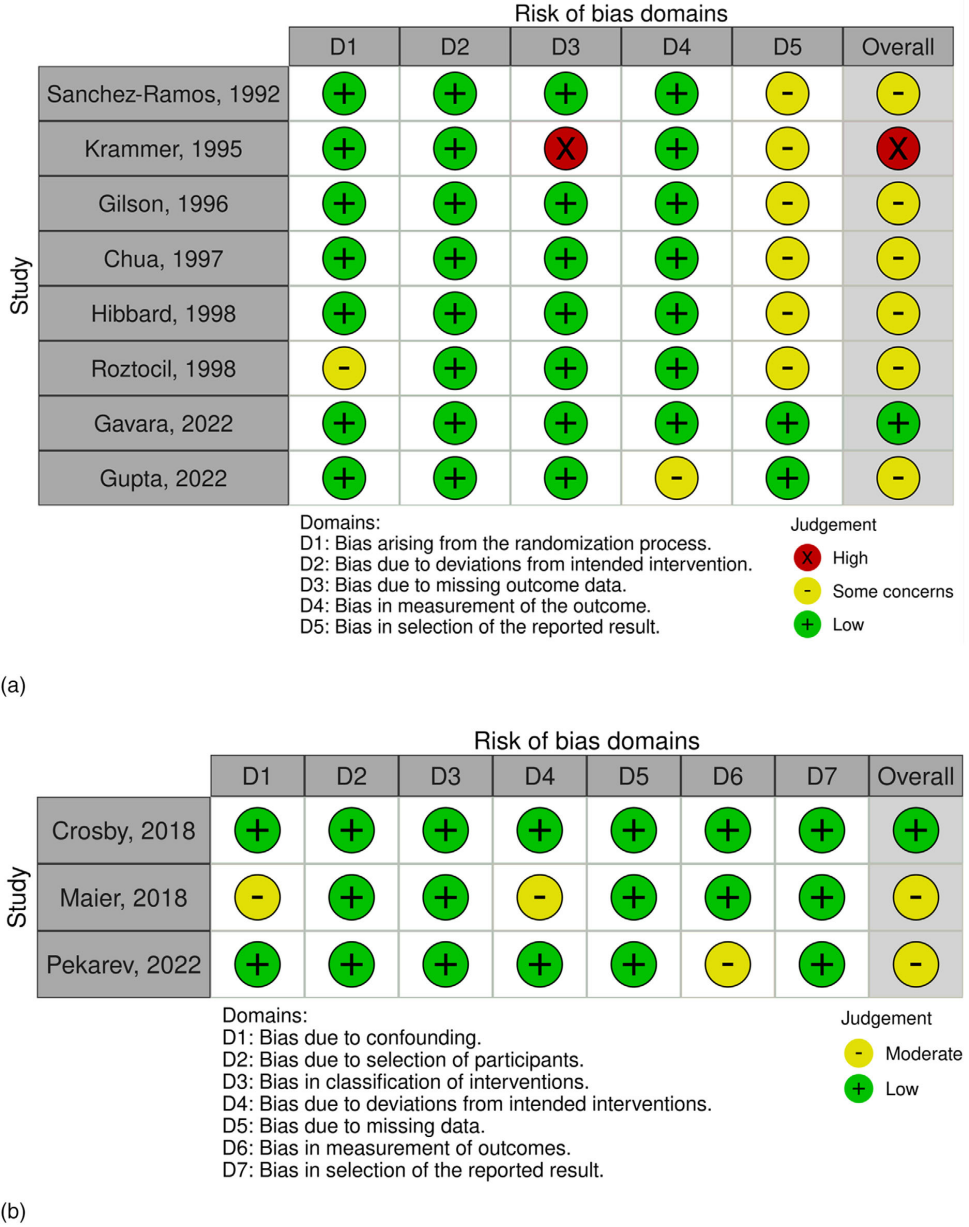
Assessment of Risk of Bias of Selected Studies (a) Randomized controlled trials. (b) Cohort studies.

Figure 2 presents the results of the quality assessment of the selected studies. The overall quality of included studies was moderate, as most RCTs (6 of 8 studies) rated as having some concerns according to ROB2,^22^, ^24^, ^29^, ^31^, ^35^, ^36^ and 2 of 3 cohort studies^33^, ^34^ were rated as having moderate risk of bias by ROBINS‐I.

Most RCTs demonstrated low risk of bias in in 4 domains: randomization (7 of 8 studies), deviations from intended intervention (8 of 8 studies), missing outcome data (7 of 8 studies), and outcome measurement (7 of 8 studies). However, concerns were identified in selective reporting, with only 2 studies^15^, ^24^ showing low risk, whereas 6 studies had moderate risk (Figure 2a).^22^, ^29^, ^31^, ^32^, ^35^, ^36^

All 3 cohort studies showed low risk in 4 domains: selection of participants, intervention classification, missing data, and selective reporting. One study showed moderate risk in confounding,^33^ deviations from intended interventions,^33^ and measurement of outcomes,^34^ whereas the other 2 showed low risk (Figure 2b).

### Maternal Safety Outcomes

Table 2 and Supporting Information: Appendix S3 present maternal safety findings from meta‐analyses examining infection, uterine hyperstimulation, postpartum bleeding, and digestive symptoms.

**Table 2:** Meta‐Analysis Outcomes of Included Studies

Outcomes	Methods (Intervention/Comparator)	Studies, n	Population (Intervention/Comparator), n	Events (Intervention/Comparator), n	Measure	Pooled Effect (95% CI)	*I^2^*, %	*P*
**Maternal Safety**								
**Maternal infection**	Synthetic osmotic dilator/PGE_2_	4	613/491	65/40	RR	1.54 (1.07‐2.21)	0	.02
	Synthetic osmotic dilator/PGE_1_	1	151/151	18/20	RR	0.9 (0.50‐1.63)	NA	.73
	Synthetic osmotic dilator + PGE_2_/PGE_2_	1	22/17	13/2	RR	5.02 (1.31‐19.33)	NA	.02
	Synthetic osmotic dilator + oxytocin/oxytocin	1	112/128	10/14	RR	0.82 (0.38‐1.76)	NA	.61
	Total	6	898/787	106/76	RR	1.27 (0.85‐1.88)	37	.24
**Uterine hyperstimulation**	Synthetic osmotic dilator/PGE_2_	4	449/506	4/26	RR	0.22 (0.09‐0.58)	0	.002
	Synthetic osmotic dilator/PGE_1_	1	151/151	35/70	RR	0.5 (0.36‐0.70)	NA	<.001
	Total	5	600/657	39/96	RR	0.45 (0.32‐0.64)	2	<.001
**Postpartum bleeding**	Synthetic osmotic dilator/PGE_2_	3	359/411	96/126	RR	0.89 (0.72‐1.10)	0	.28
	Synthetic osmotic dilator/PGE_1_	1	151/151	1/3	RR	0.33 (0.04‐3.17)	NA	.34
	Synthetic osmotic dilator + oxytocin/oxytocin	1	112/128	2/5	RR	0.46 (0.09‐2.31)	NA	.34
	Total	5	622/690	99/134	RR	0.87 (0.70‐1.08)	0	.20
**Digestive symptoms**	Synthetic osmotic dilator/PGE_2_	2	333/385	1/11	RR	0.15 (0.03‐0.84)	0	.03
**Neonatal Safety**								
**Neonatal infection**	Synthetic osmotic dilator/PGE_2_	5	703/698	86/73	RR	1.26 (0.79‐1.99)	25	.33
	Synthetic osmotic dilator/PGE_1_	1	151/151	1/3	RR	0.33 (0.04‐3.17)	NA	.34
	Synthetic osmotic dilator + oxytocin/oxytocin	1	112/128	1/0	RR	3.42 (0.14‐83.23)	NA	.45
	Total	7	966/977	88/76	RR	1.19 (0.81‐1.75)	10	.38
**1‐min Apgar score below study‐specific cutoff values**	Synthetic osmotic dilator/PGE_2_	2	108/107	2/3	RR	0.71 (0.10‐5.17)	12	.74
**5‐min Apgar score below study‐specific cutoff values**	Synthetic osmotic dilator/PGE_2_	4	231/251	7/6	RR	1.26 (0.24‐6.53)	35	.78
	Synthetic osmotic dilator/PGE_1_	1	151/151	0/1	RR	0.33 (0.01‐8.12)	NA	.50
	Synthetic osmotic dilator + oxytocin/oxytocin	1	112/128	2/6	RR	0.38 (0.08‐1.85)	NA	.23
	Total	6	494/530	9/13	RR	0.74 (0.25‐2.13)	11	.57
**Admission to NICU**	Synthetic osmotic dilator/PGE_2_	5	571/579	54/51	RR	1.05 (0.73‐1.51)	0	.78
	Synthetic osmotic dilator/PGE_1_	1	151/151	9/8	RR	1.13 (0.45‐2.84)	NA	.80
	Total	6	722/730	63/59	RR	1.06 (0.76‐1.49)	0	.72
**Perinatal death**	Synthetic osmotic dilator/PGE_2_	3	509/515	1/2	RR	0.76 (0.05‐11.42)	34	.84
**Efficacy**								
**Vaginal birth**	Synthetic osmotic dilator/PGE_2_	7	818/827	557/576	RR	0.98 (0.91‐1.06)	26	.66
	Synthetic osmotic dilator/PGE_1_	1	151/151	110/110	RR	1 (0.87‐1.15)	NA	.99
	Synthetic osmotic dilator + PGE_2_/PGE_2_	1	22/17	14/15	RR	0.72 (0.50‐1.03)	NA	.08
	Synthetic osmotic dilator + oxytocin/oxytocin	1	112/128	71/79	RR	1.03 (0.84‐1.25)	NA	.79
	Total	10	1103/1123	752/780	RR	0.98 (0.92‐1.04)	20	.50
**Change in Bishop's score**	Synthetic osmotic dilator/PGE_2_	6	755/753	NA	MD	−0.06 (‐0.36‐0.14)	86	.57
	Synthetic osmotic dilator + PGE_2_/PGE_2_	1	22/17	NA	MD	1.5 (0.53‐2.47)	NA	.003
	Total	7	777/770	NA	MD	0 (‐0.21‐0.22)	87	.97
**Additional procedures**								
Instrumental birth	Synthetic osmotic dilator/PGE_2_	6	289/305	45/41	RR	1.13 (0.77‐1.65)	0	.53
	Synthetic osmotic dilator/PGE_1_	1	151/151	6/6	RR	1 (0.33‐3.03)	NA	.99
Use of oxytocin	Synthetic osmotic dilator/PGE_2_	3	394/406	259/172	RR	1.5 (1.13‐2.00)	71	.005
Use of additional PGE_2_	Synthetic osmotic dilator/PGE_2_	1	26/26	10/4	RR	2.5 (0.90‐6.96)	NA	.08
AROM	Synthetic osmotic dilator/PGE_2_	2	368/380	256/165	RR	1.57 (1.30‐1.89)	22	<.001
	Total	13	1228/1268	576/388	RR	1.53 (1.37‐1.71)	11	<.001

Abbreviations: AROM, artificial rupture of membranes; NA, not associated; NICU, neonatal intensive care unit; MD, mean difference; PGE_1_, prostaglandin E_1_; PGE_2,_ prostaglandin E_2_; RR, relative risk.

^a^ Cutoff values for Apgar scores varied across studies, ranging from <6 to <9.

The synthetic osmotic dilator's risk of maternal infection varied by comparator. Maternal infection rate was higher when compared with PGE_2_ alone (RR, 1.54; 95% CI, 1.07‐2.21; *P =* .02) and when combined with PGE_2_ versus PGE_2_ alone (RR, 5.02; 95% CI, 1.31‐19.33; *P =* .02). However, no significant differences were found when compared with PGE_1_ (RR, 0.90; 95% CI, 0.50‐1.63; *P =* .73) or when used in combination with oxytocin versus oxytocin alone (RR, 0.82; 95% CI, 0.38‐1.76; *P =* .61). Overall, the maternal infection rate between synthetic osmotic dilators and various pharmacologic agents showed no significant difference (RR, 1.27; 95% CI, 0.85‐1.88; *P =* .24; *I^2^* = 37%).

Subgroup analyses were performed to identify differences in maternal infection according to publication year and the type of synthetic osmotic dilators (Dilapan vs Dilapan‐S). No significant differences in infection rates were observed between the synthetic osmotic dilators (Dilapan) and pharmacologic agents in studies published before 1998 (RR, 1.50; 95% CI, 0.82‐2.72; *P =* .19; *I^2^* = 54%), and between Dilapan‐S and pharmacologic agents in studies published after 2017 (RR, 0.92; 95% CI, 0.55‐1.55; *P =* .76; *I^2^* = 0%) (Supporting Information: Appendix S4).

The rate of uterine hyperstimulation was consistently lower with synthetic osmotic dilators than with PGE_2_ (RR, 0.22; 95% CI, 0.09‐0.58; *P =* .002) and PGE_1_ (RR, 0.50; 95% CI, 0.36‐0.70; *P <* .001), with the overall analysis confirming this trend (RR, 0.45; 95% CI, 0.32‐0.64; *P <* .001; *I^2^* = 2%).

The risk of postpartum bleeding with synthetic osmotic dilators was not significantly different from the risk with pharmaceutical agents, including PGE_2_ (RR, 0.89; 95% CI, 0.72‐1.10; *P =* .28) and PGE_1_ (RR, 0.33; 95% CI, 0.04‐3.17; *P =* .34). Adding osmotic dilators to oxytocin did not increase the risk of postpartum bleeding compared with oxytocin alone (RR, 0.46; 95% CI, 0.09‐2.31; *P =* .34). The overall analysis also showed no significant difference (RR, 0.87; 95% CI, 0.70‐1.08; *P =* .20; *I^2^* = 0%).

Digestive symptoms were significantly lower with synthetic osmotic dilators than with PGE_2_ (RR, 0.15; 95% CI, 0.03‐0.84; *P =* .03; *I^2^* = 0%).

### Neonatal Safety Outcomes

Table 2 and Supporting Information: Appendix S5 present findings for neonatal safety, including neonatal infection, Apgar scores, NICU admission, and perinatal death.

Neonatal infection risk with synthetic osmotic dilators was not significantly different than with pharmacologic agents overall (RR, 1.19; 95% CI, 0.81‐1.75; *P =* .38; *I^2^* = 10%). This finding was consistent in subgroup analysis by specific pharmacologic agents: PGE_2_ (RR, 1.26; 95% CI, 0.79‐1.99; *P =* .33), PGE_1_ (RR, 0.33; 95% CI, 0.04‐3.17; *P =* .34), and oxytocin alone (RR, 3.42; 95% CI, 0.14‐83.23; *P =* .45).

The risk of a 5‐minute Apgar score below the predefined cutoff (ranging from <6 to <9 across studies) was not significantly different between osmotic dilators and pharmacologic agents overall (RR, 0.74; 95% CI, 0.25‐2.13; *P =* .57; *I^2^* = 11%). This result was consistent in subgroup analysis by pharmacologic agents: PGE_2_ (RR, 1.26; 95% CI, 0.24‐6.53; *P =* .78), PGE_1_ (RR, 0.33; 95% CI, 0.01‐8.12; *P =* .50), and oxytocin alone (RR, 0.38; 95% CI, 0.08‐1.85; *P =* .23).

Similarly, NICU admission rates (RR, 1.06; 95% CI, 0.76‐1.49; *P =* .72; *I^2^* = 0%) and perinatal death risk (RR, 0.76; 95% CI, 0.05‐11.42; *P =* .84; *I^2^* = 34%) were not significantly different between synthetic osmotic dilators and pharmacologic agents.

### Efficacy Outcomes

Table 2 and Supporting Information: Appendix S6 present findings for efficacy outcomes, including vaginal birth rates, change in Bishop's score, and the need for additional procedures. Vaginal birth rates were similar between synthetic osmotic dilators and pharmacologic agents overall (RR, 0.98; 95% CI, 0.92‐1.04; *P =* .50; *I^2^* = 20%). In subgroup analyses, no significant differences were constantly observed when comparing osmotic dilators with PGE_2_ alone (RR, 0.98; 95% CI, 0.91‐1.06; *P =* .66), combined osmotic dilators and PGE_1_ with PGE_1_ alone (RR, 1.00; 95% CI, 0.87‐1.15; *P >* .99), combined osmotic dilators and PGE_2_ with PGE_2_ alone (RR, 0.72; 95% CI, 0.50 to 1.03; *P =* .08), and combined osmotic dilators and oxytocin with oxytocin alone (RR, 1.03; 95% CI, 0.84‐1.25; *P =* .79).

The change in Bishop's score with synthetic osmotic dilators was not significantly different from pharmacologic agents overall (MD, 0.00; 95% CI, ‐0.21 to 0.22; *P =* .97; *I^2^* = 87%). However, osmotic dilators combined with PGE_2_ showed a significantly greater change in Bishop's score compared with PGE_2_ alone (MD, 1.50; 95% CI, 0.53‐2.47; *P =* .003).

The need for additional procedures was significantly higher with synthetic osmotic dilators than pharmacologic agents overall (RR, 1.53; 95% CI, 1.37‐1.71; *P <* .001; *I^2^* = 11%). Comparing osmotic dilators with PGE_2_, there were no significant differences in instrumental birth (RR, 1.13; 95% CI, 0.77‐1.65; *P =* .53) and oxytocin use (RR, 1.50; 95% CI, 1.13‐2.00; *P =* .005). However, there was a significantly higher risk of AROM with osmotic dilators than with PGE_2_ (RR, 1.57; 95% CI, 1.30‐1.89; *P <* .001).

### Publication Bias

**Figure 3:**
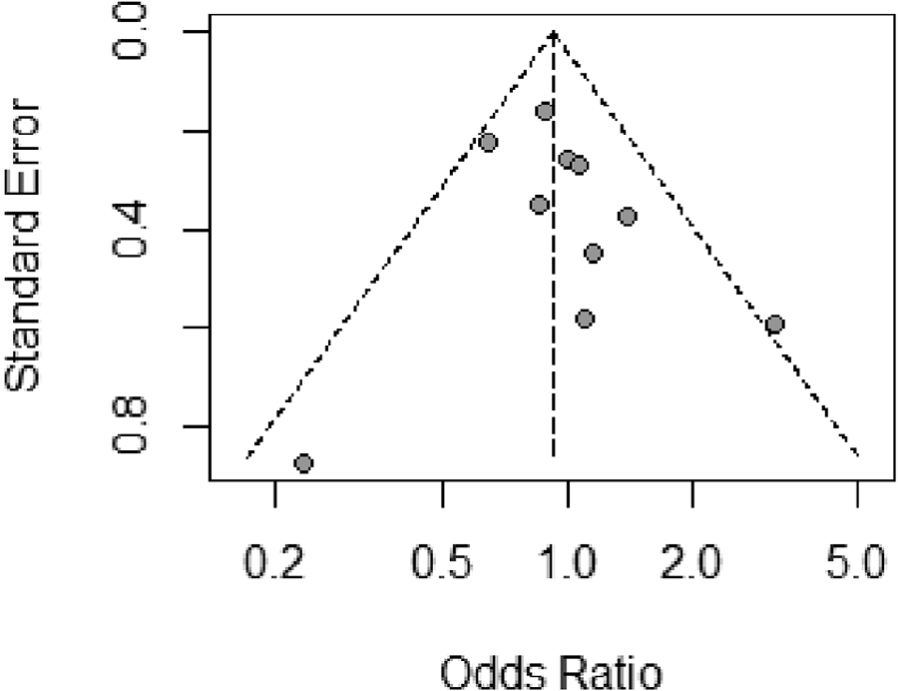
Funnel Plot for Assessment of Publication Bias

Figure 3 presents the publication bias analysis using funnel plots. The funnel plot for the rate of vaginal birth from 10 studies^15^, ^22^, ^24^, ^29^, ^30^, ^31^, ^32^, ^33^, ^35^, ^36^ showed slight asymmetry, with underreporting of studies with small effect sizes compared with studies with greater effect sizes. However, Egger's test did not suggest publication bias (Egger bias, 0.61; SE, 0.84; *P* = .487).

## DISCUSSION

This systematic review and meta‐analysis compared the safety and efficacy of synthetic osmotic dilators with pharmacologic agents for labor induction, based on 11 studies involving 2355 women for different indications. Moderate‐quality evidence shows no significant differences between the 2 methods in most safety outcomes (postpartum bleeding, neonatal infection, Apgar scores, NICU admission, and perinatal death) and efficacy outcomes (vaginal birth, Bishop score). Synthetic osmotic dilators reduced the risk of uterine hyperstimulation and digestive symptoms compared with PGE_2_ and PGE_1_. Our meta‐analysis provides robust evidence that synthetic osmotic dilators are a viable option for labor induction, demonstrating comparable safety and efficacy to pharmacologic methods while reducing the risk of uterine hyperstimulation and digestive symptoms.^24^, ^29^, ^30^, ^35^ Given the underrepresentation of synthetic osmotic dilators in major clinical guidelines, such as those of the NICE,^2^ WHO,^7^, ^27^ and ACOG,^20^ our findings offer valuable evidence to inform future guideline development.

The lower risk of hyperstimulation with synthetic osmotic dilators supports their use for cervical ripening, particularly in women with previous uterine scars (eg, prior cesarean birth), in whom PGE agents are contraindicated due to the heightened risk of uterine rupture from hyperstimulation.^33^, ^47^ This is consistent with NICE^2^ and WHO^7^, ^27^ guidelines, which suggest that mechanical methods are beneficial for avoiding uterine hyperstimulation or when PGEs are contraindicated. The favorable safety profile of synthetic osmotic dilators further suggests their potential suitability for outpatient cervical ripening, as the lower incidence of hyperstimulation may reduce the need for continuous uterine and fetal monitoring.^24^, ^30^ However, only 2 studies^35^, ^36^ in this review compared synthetic osmotic dilators with pharmacologic agents in outpatient settings. This highlights the need for further research to evaluate the safety and efficacy of synthetic osmotic dilators in outpatient settings.

Regarding safety concerns, synthetic osmotic dilators showed higher maternal infection rates than PGE_2_ alone (RR, 1.54; 95% CI, 1.07‐2.21) but similar rates compared with all pharmacologic agents (RR, 1.27; 95% CI, 0.85‐1.88). This inconsistency can be attributed to the wide publication range of included studies (1992‐2022), as only early studies from 1995 to 1998^31^, ^32^ reported higher infection rates with synthetic osmotic dilators, during a period when aseptic technique and infection control standards differed from current clinical practice. Our subgroup analyses of recent studies published after 2018^15^, ^24^, ^30^ showed similar maternal infection rates with osmotic dilators and pharmacologic agents. Despite the similar maternal infection risk, strict aseptic technique should be used during osmotic dilator insertion.^11^

Our findings with synthetic osmotic dilators were similar to systematic reviews of the safety and efficacy of Foley balloon catheters.^11^, ^12^ These reviews have shown that Foley balloon catheters are comparable to PGE_2_ and PGE_1_, with respect to vaginal birth rates and neonatal safety, while offering a reduced risk of uterine hyperstimulation. This consistent pattern across mechanical methods suggests that mechanical approaches show comparable efficacy to pharmacologic agents, with marginally increased safety regarding hyperstimulation risk.

Research on synthetic osmotic dilators was conducted between 1992 and 1998, followed by a nearly 2‐decade gap until 2018. This research trend can be explained by the FDA's approval of Dilapan‐S, a synthetic osmotic dilator, in 2015,^14^ which sparked renewed interest in this field. Our findings indicate that evidence supporting the use of synthetic osmotic dilators for cervical ripening has steadily increased since this approval. Given the growing interest in synthetic osmotic dilators^9^, ^24^ and the supporting evidence on efficacy and safety^15^, ^22^, ^24^, ^29^, ^30^, ^31^, ^32^, ^33^, ^34^, ^35^, ^36^ from this meta‐analysis, clinicians should be educated about their use as a safe and effective option for labor induction. By making synthetic osmotic dilators available, institutions can support patient choice and clinical decision‐making, and improve patient safety.

This systematic review identified that only 2 studies published in the late 1990s compared combinations of synthetic osmotic dilators with pharmacologic agents,^22,^ ^31^ with no recent studies examining these combinations. Combinations of the Foley balloon catheter and pharmacologic methods have demonstrated improved cervical maturation and reduced length of labor by 3 to 4 hours compared with single‐agent methods.^34^, ^48^ Given the limited evidence but potential benefits of these combination approaches, further research investigating synthetic osmotic dilators combined with pharmacologic agents is needed to optimize labor induction protocols.

Several limitations warrant consideration. The included studies span 3 decades (1992‐2022), with older studies potentially not reflecting current practices. Furthermore, heterogeneity existed in study characteristics, including participant factors (gestational age, parity, Bishop score), intervention methods (number and duration of dilators), and comparator details (type, dosage, and administration routes of pharmacologic agents), necessitating cautious interpretation of results. Moreover, we could not assess the effects of combining synthetic osmotic dilators with pharmacologic agents due to the limited number of studies. Finally, restricting to English‐language studies may have excluded potentially eligible articles.

## CONCLUSION

This systematic review and meta‐analysis of 11 studies suggests that synthetic osmotic dilators have comparable efficacy and maternal and neonatal safety to pharmacologic agents (PGE_1_, PGE_2_, and oxytocin) for cervical ripening in labor induction, with advantages in reducing uterine hyperstimulation and digestive symptoms. These findings support synthetic osmotic dilators as an effective and safe cervical ripening method, particularly when pharmacologic agents are contraindicated, such as in women with previous cesarean birth due to hyperstimulation‐associated rupture risk. Study findings could inform the integration of synthetic osmotic dilators into international guidelines for cervical ripening and induction of labor.

## CONFLICT OF INTEREST

The authors have no conflicts of interest to disclose.

## Supporting information




**Appendix S1**. PRISMA 2020 Checklist
**Appendix S2**. Database Search Terms
**Appendix S3**. Forest Plot for Maternal Safety Outcomes
**Appendix S4**. Subgroup Analysis of Maternal Infection by Publication Year
**Appendix S5**. Forest Plot for Neonatal Safety Outcomes
**Appendix S6**. Forest Plot for Efficacy

## Data Availability

The data that support the findings of this study were extracted from published articles listed in our references. The extracted data are available from the corresponding author upon reasonable request.
